# Supplementary figures and images for: A Transcriptomic and Metabolomic Study on the Biosynthesis of Iridoids in Phlomoides rotata from the Qinghai–Tibet Plateau
Source: Plants (Basel). 2024 Jun 12;13(12):1627. doi: 10.3390/plants13121627 (PMC11207590; doi:10.3390/plants13121627)

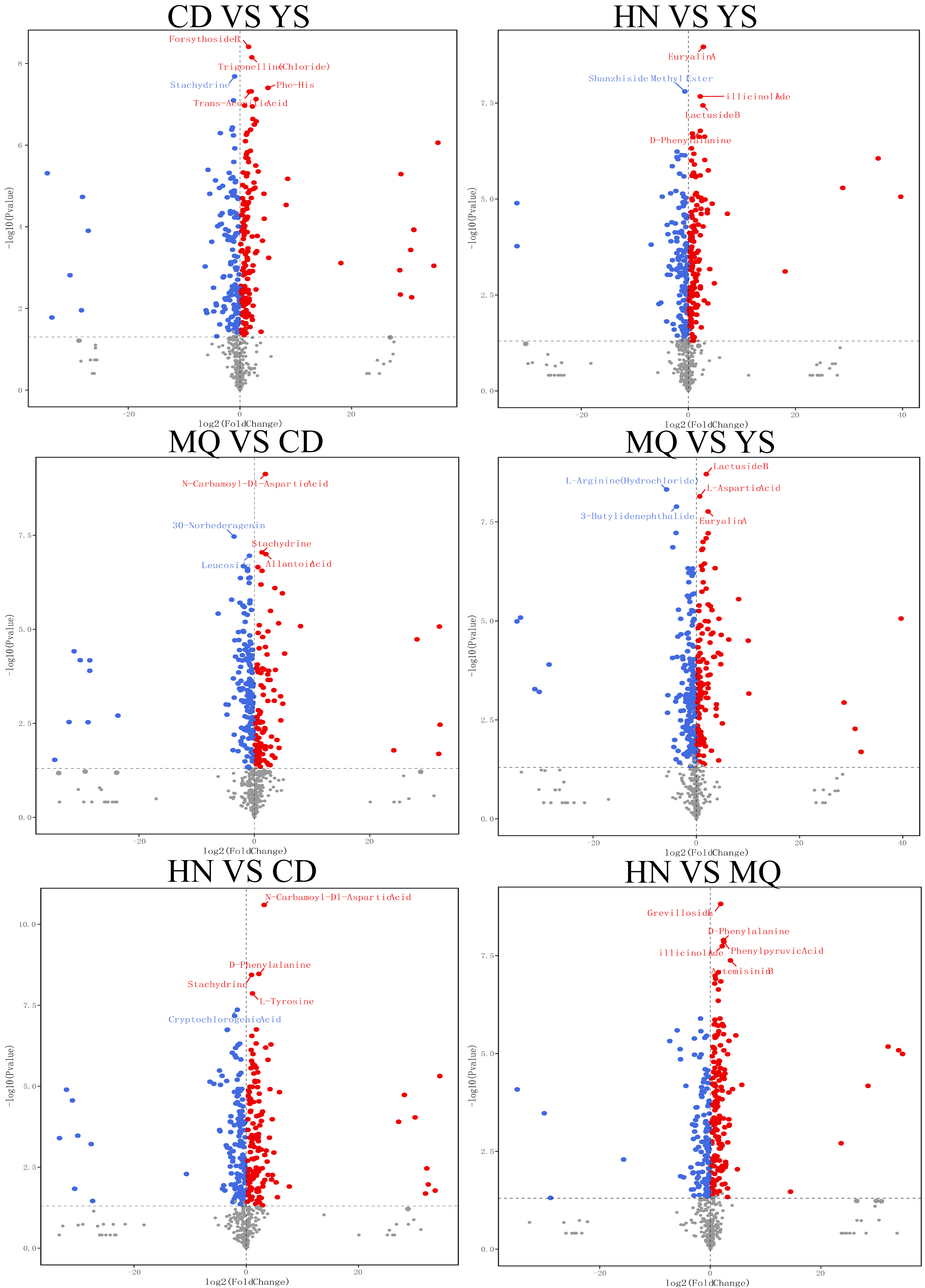

Supplement: Supplementary file 1 [file plants-13-01627-s001.zip › Fig S1.tif]

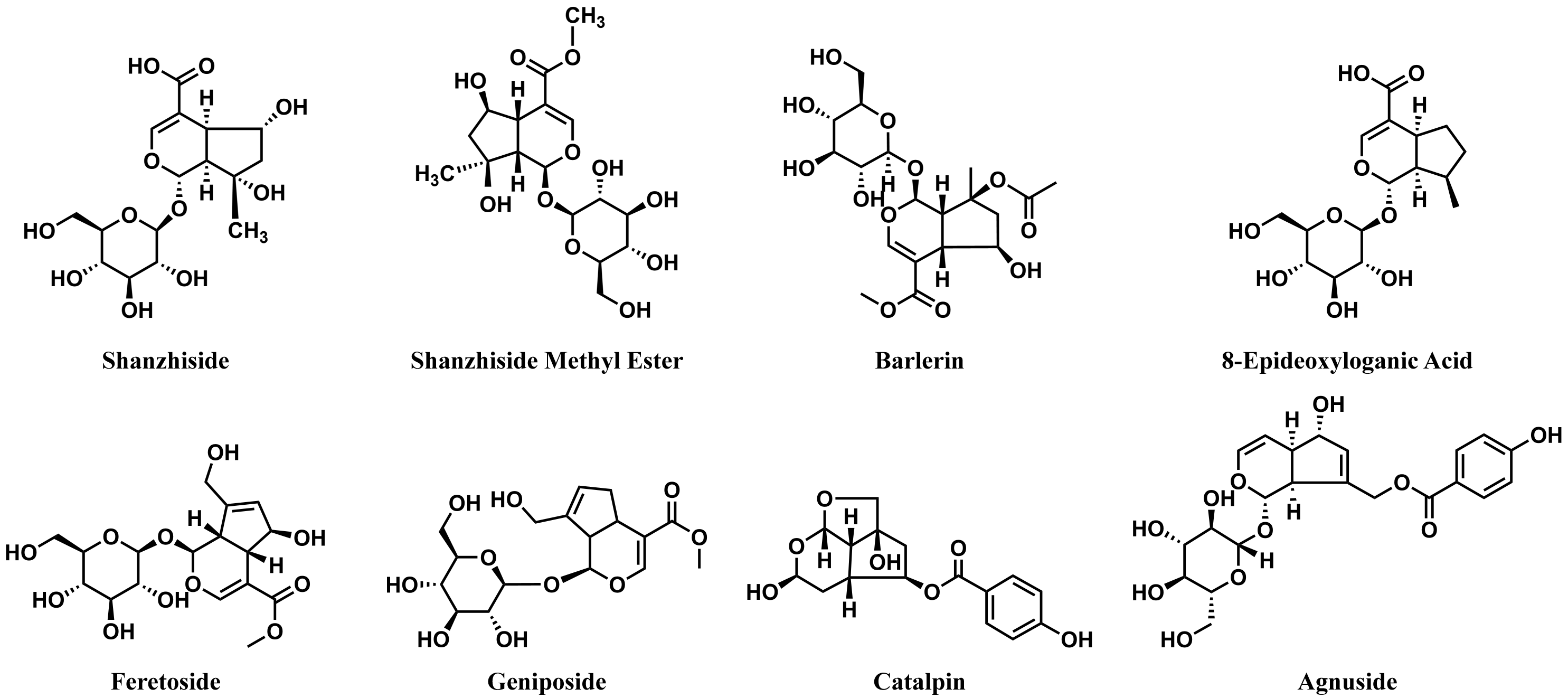

Supplement: Supplementary file 1 [file plants-13-01627-s001.zip › Fig S2.tif]

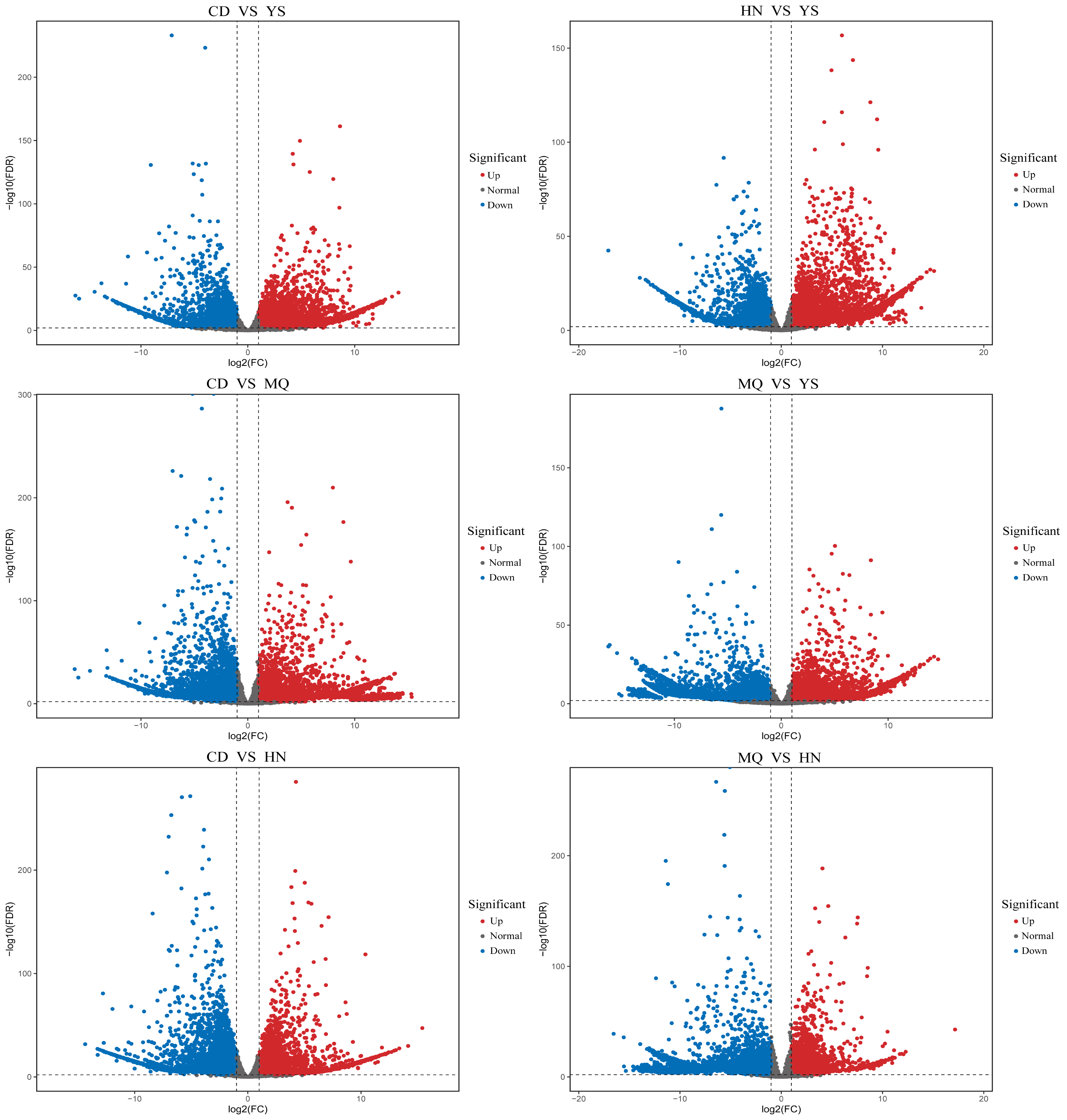

Supplement: Supplementary file 1 [file plants-13-01627-s001.zip › Fig S3.tif]

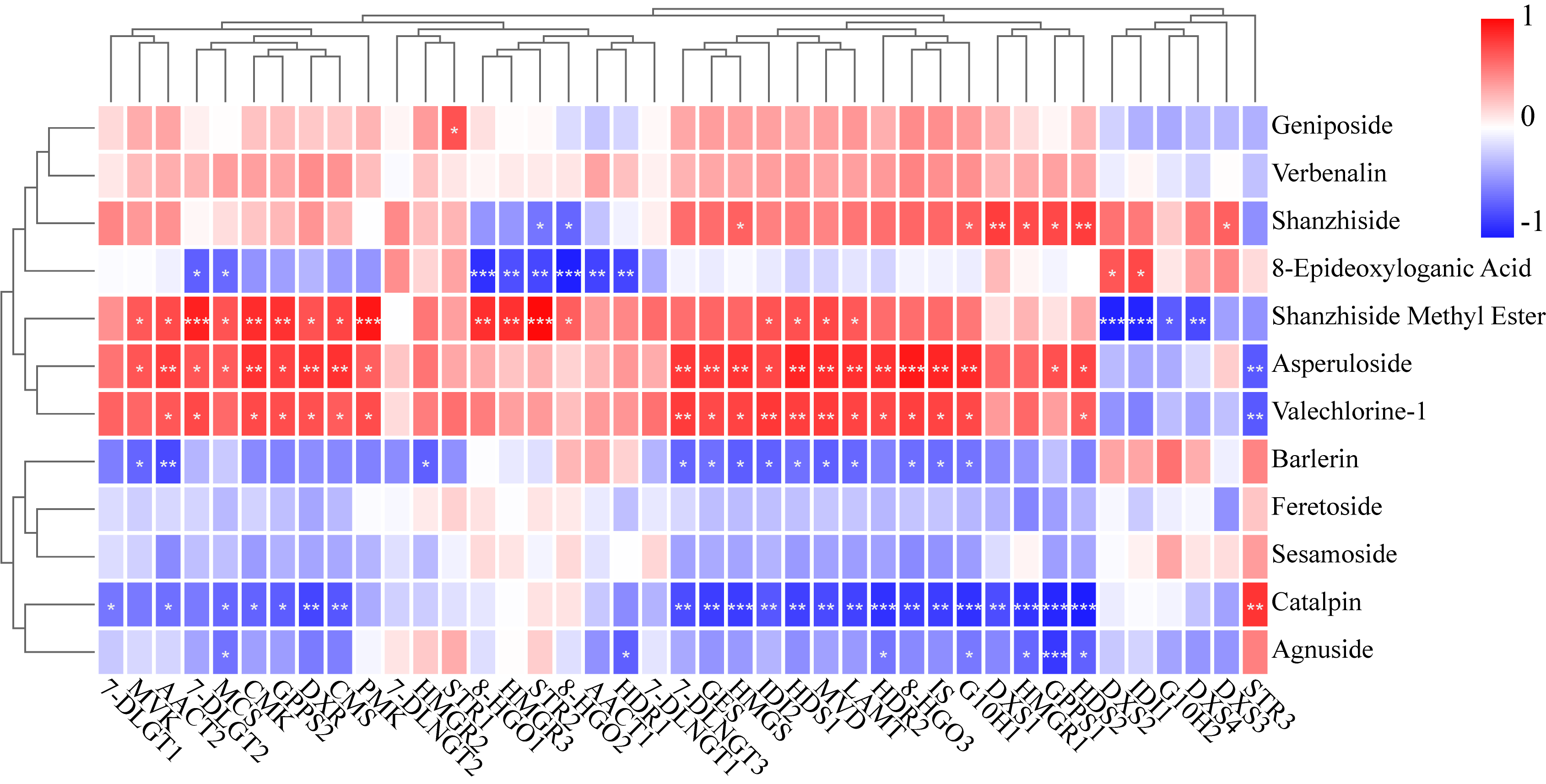

Supplement: Supplementary file 1 [file plants-13-01627-s001.zip › Fig S4.tif]
